# Cycle-Triggered Cortical Stimulation during Slow Wave Sleep Facilitates Learning a BMI Task: A Case Report in a Non-Human Primate

**DOI:** 10.3389/fnbeh.2017.00059

**Published:** 2017-04-13

**Authors:** Irene Rembado, Stavros Zanos, Eberhard E. Fetz

**Affiliations:** ^1^Department of Physiology and Biophysics and Washington National Primate Research Center, University of WashingtonSeattle, WA, USA; ^2^Center for Sensorimotor Neural Engineering (NSF ERC), University of WashingtonSeattle, WA, USA

**Keywords:** sleep, oscillations, BMI, memory consolidation, synaptic plasticity

## Abstract

Slow wave sleep (SWS) has been identified as the sleep stage involved in consolidating newly acquired information. A growing body of evidence has shown that delta (1–4 Hz) oscillatory activity, the characteristic electroencephalographic signature of SWS, is involved in coordinating interaction between the hippocampus and the neocortex and is thought to take a role in stabilizing memory traces related to a novel task. This case report describes a new protocol that uses neuroprosthetics training of a non-human primate to evaluate the effects of surface cortical electrical stimulation triggered from SWS cycles. The results suggest that stimulation phase-locked to SWS oscillatory activity promoted learning of the neuroprosthetic task. This protocol could be used to elucidate mechanisms of synaptic plasticity underlying off-line learning during sleep and offers new insights into the role of brain oscillations in information processing and memory consolidation.

## Introduction

One of the main applications of closed-loop bidirectional brain-machine interfaces (BBMI) is to generate synaptic plasticity in the central nervous system (Fetz, 2015). At the cellular level the synaptic connections between two sites can be strengthened by using spike activity recorded at one site to trigger electrical stimulation on the other site (Jackson et al., 2006; Rebesco et al., 2010; Nishimura et al., 2013). This Hebbian paradigm for creating synaptic plasticity relied on action potentials of single units and was implemented with intracortical electrodes to record neural activity and to deliver stimuli. A recent study (Zanos et al., 2016) showed that short-term changes in effective intracortical connectivity could be obtained through electrical cortical surface stimulation (ECS) triggered by brain oscillations recorded with electrocorticography (ECoG). Oscillatory activity has been implicated in information processing and consolidation of information by synchronizing neural activity at multiple temporal and spatial scales (Engel et al., 1991a,b; Buzsáki and Draguhn, 2004; Buzsáki et al., 2004). In particular, slow-wave sleep (SWS), characterized by large delta waves (1–4 Hz) mostly occurring during non-rapid-eye-movement (NREM) sleep, are crucial for learning and memory consolidation. A growing body of literature has provided important insights into its role in improving performance and in coordinating a hippocampo-cortical interaction (Ghilardi et al., 2000; Stickgold et al., 2000a,b; Walker et al., 2002, 2003a,b; Huber et al., 2004; Gulati et al., 2014; Maingret et al., 2016). SWS generated within the neocortex reflects widespread synchronization of network activity and its up and down states correspond, respectively, to widespread membrane depolarization with increased neuronal excitability, and membrane hyperpolarization with neuronal quiescence (Steriade et al., 1993b; Steriade and Timofeev, 2003; Steriade, 2003; Crochet et al., 2006). This characteristic rhythmic activity is thought to promote the consolidation of motor skills (Huber et al., 2006; Tamaki et al., 2013) and is also implicated in the consolidation of neuroprosthetic skills (Gulati et al., 2014). Therefore during SWS, synaptic connections between neuronal ensembles involved in the newly acquired skills undergo plasticity. While different hypotheses involve the mechanism which relates SWS and cortical plasticity (Tononi and Cirelli, 2003, 2006; Mölle et al., 2004; Euston et al., 2007), the role of sleep in learning and memory has yet to be precisely characterized.

One method to dynamically manipulate cortical plasticity and investigate the role of SWS in memory consolidation is by delivering electrical stimuli to the cortex during sleep and evaluating the task performance on the next day. In this context, Marshall et al. (2006) showed an enhancement in the retention of hippocampus-dependent declarative memories by inducing slow oscillations through transcranial application of oscillating potentials during SWS in humans. Maingret et al. (2016), instead, delivered pulses of electrical stimulation to the neocortex triggered by sharp-wave ripples during SWS to boost the coupling between the hippocampus and the cortex in rats being trained in a spatial memory task. This resulted in a subsequent increase in performance on the next day, compared to the control rats which performed at chance levels. Based on these observations, we developed a new protocol to investigate neuronal dynamics during sleep that employs neuroprosthetic training during wakefulness and closed-loop ECS during sleep in non-human primates. A macaque monkey was concurrently trained in two brain-machine interface (BMI) tasks of equal difficulty; SW cycle-triggered stimulation during sleep was delivered to the cortical site involved in one of the two BMI tasks. We timed the stimulation to occur in the up-state of SWS. Gulati et al. (2014) reported that task-related neurons, whose rate the animals learned to enhance during a BMI task, increased their entrainment to the up-state of SWS after learning, with no consistent change in the local field potential (LFP) power spectrum. These up-states are an indirect mark of correlated activity in large neuronal populations and the Gulati et al. (2014) study showed they play role in memory consolidation. We wanted to test this hypothesis, by coupling the electrical stimulation with the negative phases of SWS, which are signatures of up-states. Our hypothesis is that enhancement of SWS activity through precisely-timed electrical stimulation would promote a temporal “rearrangement” of neural activity involved in the task which could help consolidate synaptic plasticity. Here we showed that the BMI task, whose cortical site was associated with cortical stimulation during SWS, was easily recalled and was performed at a faster rate. Given the nature of the case report and pending relevant control experiments, we believe this study can serve as proof-of-principle that the behavioral consequences of closed-loop stimulation during sleep can be studied using a BMI task. Such studies may elucidate mechanisms of cortical plasticity associated with memory consolidation and off-line learning.

## Materials and Methods

Experiments were performed with a male macaque nemestrina monkey (12 Kg). The experiments were approved by the Institutional Animal Care and Use Committee (IACUC) at the University of Washington and all procedures conformed to the National Institutes of Health Guide for the Care and Use of Laboratory Animals.

### Cortical Implant

The implant surgery was performed using sterile techniques while the animal was anesthetized with sevoflurane gas. Epidural electrodes were implanted through individual 0.5 mm burr holes drilled with a stereotaxic guide. A total of 24 electrodes were placed in two hemispheres. On each hemisphere, nine electrodes were located over the sensorimotor cortex (primarily M1), arranged in a 3 × 3 grid with 3-mm spacing, and three electrodes over the supplementary motor cortex (SMA). The epidural electrodes were made with 9 mm cut length of platinum rod (AM Systems #711000, 0.254 mm diameter) insulated with heat-shrink Pebax (Small Parts #B0013HMWJQ). Pebax was cut so that the diameter of the exposed tip was ~0.5 mm, corresponding to an exposed surface area of ~0.06 mm^2^. Skull screws placed in the occipital area served as ground leads. The implant and the connectors were secured to the skull with acrylic cement and enclosed in a titanium casing that was also attached to the skull with cement and skull screws.

### EOG Implant

In a second surgery the monkey received electrooculography (EOG) electrodes. After skin incision, insulated stainless steel wires were secured to the bone with titanium screws and were routed subcutaneously to a connector in the chamber. EOG leads were placed at the lateral can thus and superior margin of the left orbit; another lead was positioned at the superior margin of the right orbit.

### Experimental Timeline

To test the hypothesis that off-line learning can be influenced by sleep-related stimulation, we trained the monkey to perform two BMI tasks of equal difficulty; one was associated with ipsilateral stimulation, the other was not. Each BMI task involved the volitional control of oscillatory ECoG activity at two selected motor cortical sites, one on the left hemisphere (“left task”) and the other on the right hemisphere (“right task”). The monkey was trained on both tasks daily, during two 2-h-long sessions, one in the morning and one in the evening, for a total of 17 days. The daily training order for the two tasks was randomized between morning and evening times, to control for the effect of time of day on learning (Kemény and Lukács, 2016). Between training sessions the monkey was returned to the cage.

**Figure 1:**
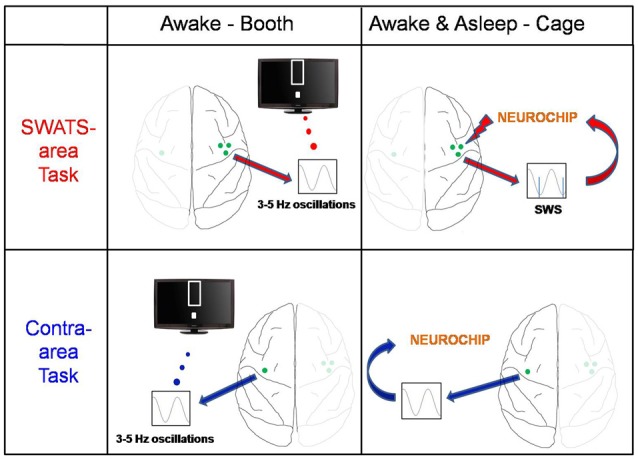
**Experiment schema.** The monkey was trained on two brain-machine interface (BMI) tasks: the slow-wave activity triggered stimulation (SWATS)-area task (top panel) was paired with slow-wave-triggered stimulation after each training session, while the contra-area task (bottom panel) had no associated stimulation. The monkey had to learn to enhance low-frequency oscillations through one M1 electrode; the only difference between the two tasks was the hemisphere: while SWATS-area task involved M1 electrode of right hemisphere, contra-area task involved an M1 electrode of left hemisphere. For SWATS-area task, slow-waves triggered stimuli were delivered on two electrodes adjacent to the trained electrode through Neurochip2 when the monkey was housed in the cage after the training in booth.

After the end of the evening training session the monkey was returned to the cage for the night. Using a portable computer for recording and stimulation, the Neurochip2 (Zanos et al., 2011), stimulation triggered by low frequency oscillatory ECoG activity during sleep was delivered to the right site. We will refer to the right task as the “slow-wave activity triggered stimulation” (SWATS) task for the rest of manuscript. The left site did not receive any stimulation (“contra-area” task; Figure 1). Sleep-related stimulation occurred every night for the duration of the 17 days of BMI training.

### Booth Recording

In the booth, ECoG activity was recorded from the epidural electrodes using active headstages (ZC-16, Tucker-Davis, Alachua, FL, USA) and digital amplifiers (gUSBamp, g.tecMedical Engineering Gmbh, Schiedlberg, Austria). Signals were sampled at 2.4 kS/s. Data from the amplifiers were streamed to a personal computer through a USB link, then stored and visualized in real-time using a Simulink-based (MathWorks, Natick, MA, USA) graphical user interface (GUI), developed in-house.

### Brain-Control Task

During BMI training the monkey had to control a computer cursor using volitionally controlled ECoG activity from a selected cortical electrode. The cortical signal was filtered in real-time between 2–5 Hz (delta band), and the power was linearly converted to the vertical position of a cursor on a video monitor placed in front of the monkey. Specifically, the algorithm implemented the sliding Discrete Fourier Transform (DFT) where the spectral value of the *k*th bin is calculated from N-points DFT. The N-points window is then advanced by one sample in order to obtain a spectral bin output rate equals to the data input rate (Jacobsen and Lyons, 2003). Each trial began with the cursor at the bottom of the screen; the target was presented at the top. The monkey had to enhance delta activity to reach the target and hold the cursor inside the target for 0.3 s to receive a juice reward. A reward tone was delivered at the end to strengthen the association between successful trials and reward. Auditory feedback was continuously provided; tone frequency increased with the decreasing distance between the cursor and the target. The training time called “feedback-on” time, generally lasted between 40 min and 55 min per session. Each session also comprised 5–10 min of “feedback-off” time during which the display, the auditory feedback and rewards were turned off, while ongoing brain activity was still recorded. This procedure allowed us to quantify the number of spontaneous events that would correspond to “successes” which occurred while the animal was not actively performing the volitional control task. We then compared such number with the corresponding number of successes achieved during the active control (i.e., feedback-on) during the same training session.

### Recording during Sleep

Recordings during sleep occurred with the animal housed in its cage. Neurochip2 acquired three different signals at 8-bit resolution. To quantify gross motor movement (head and whole body movement), a 3-axis accelerometer powered by a 3 V lithium coin cell was fixed in the titanium casing. The three analog outputs of the accelerometer were passed through a sum-of-absolute circuit to provide acceleration magnitude. This voltage was recorded on one of Neurochip2 channels at 2 kS/s. A second recording channel recorded ECoG from one of the implanted electrodes in the motor cortex, and a third channel recorded EOG from one eye. The latter two channels were sampled at 2 kS/s.

Each recording began with the animal seated in a primate chair in the lab. Neurochip2 was then programmed by entering the desired settings into Matlab GUI and uploading them via IR connection. The animal was then returned to its cage where it moved freely until the following training session. Recorded data were stored on a removable flash memory card with 2-GB capacity and later imported to Matlab.

### Closed-Loop Stimulation during SWS

**Figure 2:**
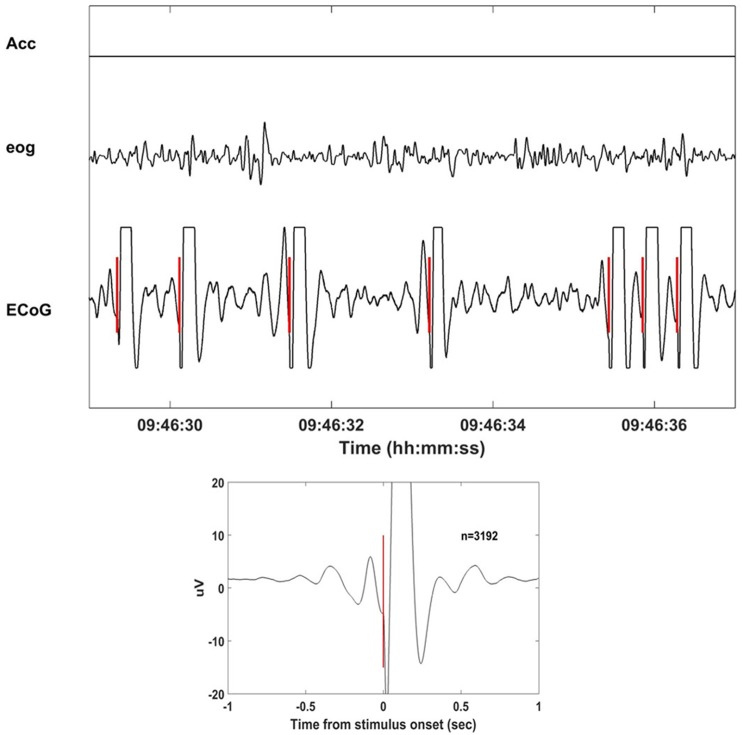
**Stimulation delivered at the slow wave sleep (SWS) troughs with Neurochip2.** Eight seconds of recorded signals (acceleration, electrooculography (EOG), electrocorticography (ECoG)) during stimulation of SWS cycles. Neurochip2 was set to deliver a single 4 mA bipolar current pulse at the trough of low-frequency-filtered local field potentials (LFPs) based on amplitude and timing of two discriminating windows. In the box below the stimulation-triggered-average over 2 h of recording (number of events = 3192). The plot shows 2 s of averaged signal centered at the stimulation onset.

Neurochip2 was programmed to discriminate the trough (surface-negative phase) of high-amplitude slow-wave oscillations from the site used for the BMI task (motor cortex of the right hemisphere) and trigger the stimulator. The discrimination was implemented via a threshold and two time-amplitude windows applied on the ongoing ECoG signal band-pass filtered in the frequency range of 1–4 Hz. The two time-amplitude windows were set to discriminate signals within the amplifier range. This prevented the discrimination of stimulus artifacts that caused saturation of the signal (Figure 2). The acceleration signal was used to gate the delivery of stimuli. To deliver the stimulation two conditions had to be met: (1) acceleration had to equal zero; and (2) ECoG signal from the recorded electrode exhibited high-amplitude, low-frequency oscillations. These two conditions allowed successful discrimination between NREM and rapid eye movement (REM) sleep in our recordings. Other studies employed neural activity together with the electromyographic activity (EMG) to score the sleep stages (Louis et al., 2004; Brankack et al., 2010; Oishi et al., 2016). The power of the neural signals is generally used to distinguish between NREM and REM, while the EMG improves the separation of waking and REM sleep. Because we did not have access to any EMG signal, we used head acceleration as a marker of movements. Neurochip2 was then configured to discriminate only delta oscillations with high amplitude; the amplitude of delta oscillations during REM sleep was too small to be detected by the discriminator. The EOG was recorded only as control signal for off-line visual inspection. The discriminator generated triggers which were used in real-time to trigger the delivery of single biphasic pulses at 4 mA to a pair of cortical electrodes placed in the close proximity to the recorded ECoG site.

### Statistical Analysis

In order to evaluate the task performance, we segmented each SWATS-area and contra-area task session in bins of 1 min duration; for each bin we counted the number of successful target acquisitions (rate of target acquisition, RTA). We then obtained, for each session, a distribution of RTA achieved during feedback-on and during feedback-off.

To test for a statistically significant difference in the RTA between feedback-on and feedback-off times within a given session, we used the *t*-test, Bonferroni-corrected for multiple comparisons (the total number of sessions). To test for a statistically significant difference in the RTA between a SWATS-area and a contra-area task on a given day, we used *t*-test, Bonferroni-corrected for multiple comparisons (the total number of days). All *t*-test were performed on MATLAB using the ttest2 function of the Statistics and Machine Learning Toolbox.

To test whether the RTA increases with training independent of the effect of the task and the feedback, we constructed a general linear model (GLM) to capture the dependence of the RTA(*Y*) on three independent variables: day (*X*_1_), task (SWATS-area or contra-area; *X*_2_) and feedback (feedback-on or feedback-off; *X*_3_):

$$Y\;=\;B_{0}+B_{1}X_{1}+B_{2}X_{2}+B_{3}X_{3}.$$

In addition to the individual coefficients *B*, the model returns a *p* value for each coefficient, which indicates the statistical significance of the contribution of the corresponding independent variable to the estimated prediction. The GLM analysis was performed on MATLAB using the generalized linear regression model function of the Statistics and Machine Learning Toolbox.

## Results

### Discrimination of NREM Slow Oscillations

**Figure 3:**
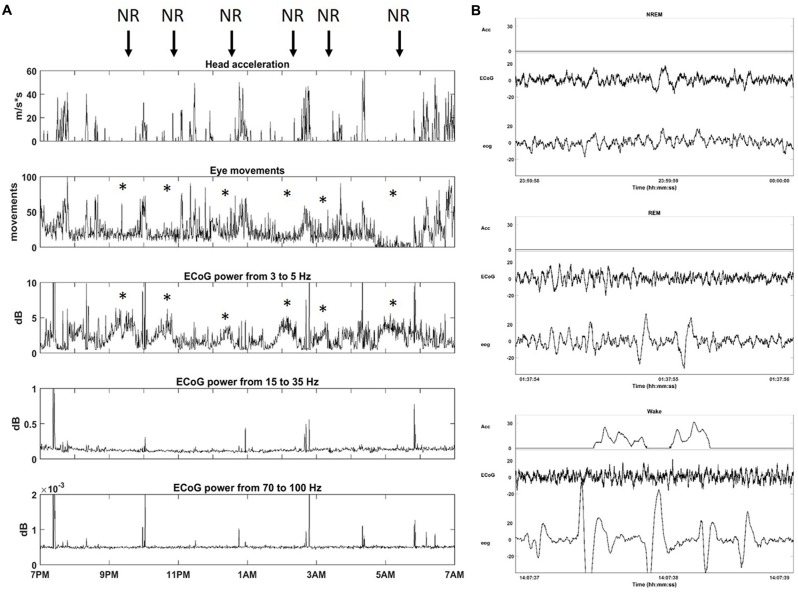
**Sleep architecture of monkey. (A)** One night of continuous recording of sleep from 7 pm to 7 am with Neurochip2. From top to bottom: head acceleration, eye movements, power (dB) for different frequency bands: Delta (3–5 Hz), Beta (15–35 Hz), Gamma (70–100 Hz). Each plot shows the average over 30-s bins. The arrows point at non-rapid-eye-movement (NREM) sleep stages (NR) characterized by a relative higher contribution of delta oscillations to ECoG signals with a reduction in eye movements (*). **(B)** Example of 2-s epoch polygraph records showing, from top to bottom, NREM, rapid eye movement (REM) and Wake stage in the monkey.

In order to configure Neurochip2 to detect SWS, we first investigated the physiological signatures of different stages of sleep in the animal. We recorded broad-band ECoG, EOG and head/whole body acceleration while the monkey was housed in its cage for 24 h at a time. Visual inspection of the signals revealed standard sleep stages previously described in Rhesus monkeys (Daley et al., 2006; Hsieh et al., 2008). Wakefulness was characterized by high-frequency, low-amplitude ECoG with large and REMs often associated with head/whole body movements (Figure 3). Sleep included two distinguishable stages: NREM and REM sleep (Figure 3). NREM sleep was characterized by absence of movement (i.e., acceleration equals zero), with high-amplitude, low-frequency ECoG and slow, reduced EOG activity. REM sleep was characterized by absence of movement, desynchronized, low-amplitude ECoG, similar to that of wakefulness and REMs (Figure 3).

**Figure 4:**
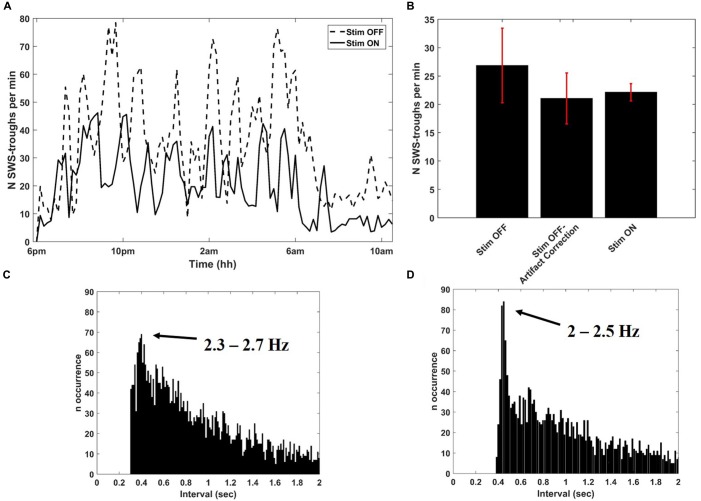
**Detection of SWS cycles from SWATS-area (with stimulation ON) and from contra-area (with stimulation OFF). (A)** SWS cycles detected per minute as a function of night time during stimulation OFF (dotted line) and stimulation ON (solid line) collected in two different nights. **(B)** Average (mean ± STD) rate of identified SWS cycles during the entire night over several recordings (*N* = 11) for three different conditions: Stimulation OFF, Stimulation OFF with artifact correction, Stimulation ON. The distributions are not significantly different (pair-wise *t*-test returned *p*-values > 0.05 for all comparisons). **(C)** Inter-discrimination- interval histogram of 2 h of recording from contra-area during sleep with deletion of 300 ms after each detected cycle that would have triggered the stimulation. The peak is centered at 0.4005 s, which corresponds to 2.5 Hz. **(D)** Inter-stimulus-interval histogram from 2 h of SWATS-area sleep recording with stimulation ON from 9 pm to 11 pm. The peak is centered at 0.4485 s, which corresponds to 2.2 Hz.

After collecting several baseline sleep recordings, we configured Neurochip2 to detect the trough of high-amplitude SWS oscillations, which mostly occur during NREM sleep (Figure 3). As expected, the number of SWS oscillations discriminated by the Neurochip3 varied throughout the night (Figure 4A), showing a pattern consistent with the time course of SWS stages during sleep. The same time course of SWS stages was also observed during sessions without stimulation, confirming that stimulation did not alter the periodic nature of REM and NREM sleep, although the rate of detected slow waves with stimulation is lower compared to that during no stimulation (Figure 4A). This difference could be due to the stimulation artifact, which saturates the signal for around 300 ms. In order to verify this hypothesis, we configured the Neurochip to detect the SWS without delivering any stimulation and we implemented a refractory period of 300 ms after each detected cycle, during which no new cycles could be detected. Figure 4B shows the distributions of the discriminated SWS oscillation rate over several recordings (*N* = 11) for three different conditions: Stimulation OFF, Stimulation OFF with simulated artifact removal and Stimulation ON. The *t*-tests returned non-significant *p*-values (*p*-values > 0.05) for all pair-wise comparisons. The simulated inter-stimulus interval histogram (Figure 4C) shows a peak around 0.4 s (2.5 Hz) which is similar to the one shown in the inter-stimulus interval histogram of Figure 4D, generated from a sleep recording with stimulation ON. In both cases the peaks are consistent with the period of slow waves.

In order to assess whether the stimulation rate showed differences between wakefulness and sleep, two representative sleep recordings, one with stimulation ON and the other with stimulation OFF, were visually scored in 30-s epochs as either sleep or awake according to the criteria established for human sleep by Rechtschaffen and Kales (1968). By counting the number of stimuli per each epoch, we estimated the rate of occurrence of slow oscillations for the two scored conditions. The same counting was performed on recording with stimulation OFF corrected by the simulated refractory period of 300 ms. The rate of detected cycles during wakefulness was significantly lower (*p*-value < 0.01) than during sleep in all three conditions, meaning that the stimulation mostly occurred during sleep (Table 1). Therefore the presence of delivered stimuli did not affect the characteristic asymmetry between wakefulness and sleep (Table 1).

**Table 1:** **Number of detected slow wave sleep (SWS) episodes per 30-s epoch (mean ± STD)**.

	Wake	Sleep
Stim ON	5.99 ± 2.64	13.97 ± 2.78
Stim OFF	10.45 ± 2.99	21.89 ± 7.98
Stim OFF-simulated artifact correction	8.34 ± 2.02	16.03 ± 5.20

### Increase of Performance for the SWATS-Area Task

**Figure 5:**
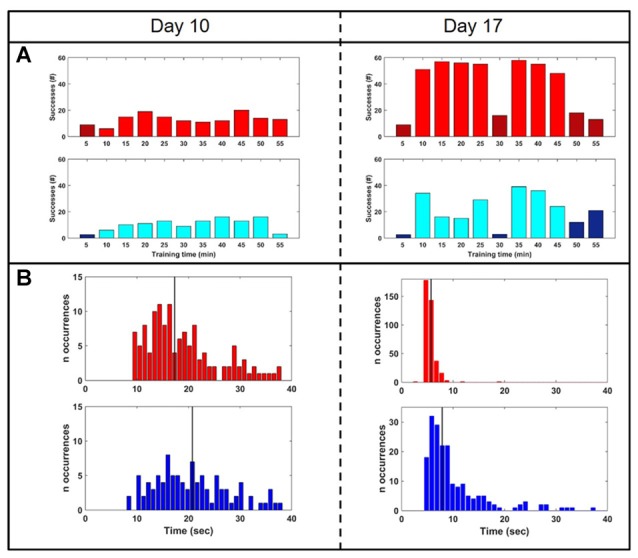
**Performance comparison: day 10 (left column) vs. day 17 (right). (A)** Rate of successes during training for SWATS-area task (top panel, red) and contra-area task (bottom panel, blue). Each bar reports the number of successes achieved in 5 min of training. Bright color corresponds to feedback-on time and dark color represents feedback-off time. **(B)** Reward interval distribution during feedback-on time for SWATS-area task (top panel, red) and contra-area task (bottom panel, blue). Black lines indicate median values.

**Figure 6:**
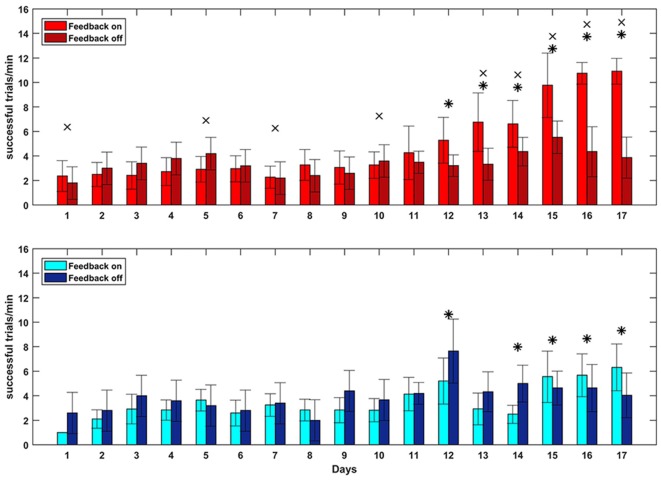
**Brain Control performance overall 17 days of training.** SWATS-area task (top panel, red), contra-area task (bottom panel, blue). Each bar represents the average number of successful trials achieved per minute (mean ± STD). Bright color shows the performance during feedback on time and dark color represents the feedback off time for each day session (*x*-axis). For both tasks, the * indicates the days when the difference in performance between feedback-on and feedback-off is significant (*p*-value < 0.05, Bonferroni corrected). The × indicates significant (*p*-value < 0.05, Bonferroni corrected) difference during feedback-on performance between the two tasks.

To test whether closed-loop stimulation during SWS affected the learning rate of a BMI task, we trained the monkey on two BMI tasks of equal difficulty, one of which (the “SWATS-area task”) was dependent on activity in the right, stimulated, hemisphere. The two tasks were trained daily, for the same amount of time, every day for 17 days. Over the course of a typical 55-min practice session, the animal showed improvements in task performance from day 10 to day 17 for both tasks, with an increase in the number of successful trials and a significant reduction in time between two consecutive rewards (Figure 5). Figure 6 shows performance in both tasks during feedback-on and feedback-off times expressed as rate of successful trials achieved per minute for each training session. Feedback-on performance in the SWATS-area task became significantly (*p* < 0.05, Bonferroni corrected) higher than feedback-off performance at day 12 (indicated with “*” in Figure 6), although the increasing trend started on day 11. For the contra-area task feedback-on performance became significantly higher than feedback-off at day 15. The feedback-on performance of the SWATS-area task became significantly higher than the same-day feedback-on performance of contra-area task starting from day 13 (indicated with “+” in Figure 6). The same *t*-test was performed to compare the performance of each day during feedback-off between the two tasks and only at day 12 the test returned a significant *p*-value (data not shown).

We then performed the GLM analysis to predict the rate of successful trials per minute (dependent variable) in term of three experimental variables: day, task and feedback-on/off time. The analysis returned small *p*-values for all three variables (respectively: *p*-value = 0, *p*-value = 3.14e-147, *p*-value = 7.75e-131. 4896 degrees of freedom), meaning that all of those were significantly affecting the estimation of the dependent variable.

### The Power of Oscillations Does Not Explain the Performance Improvement

**Figure 7:**
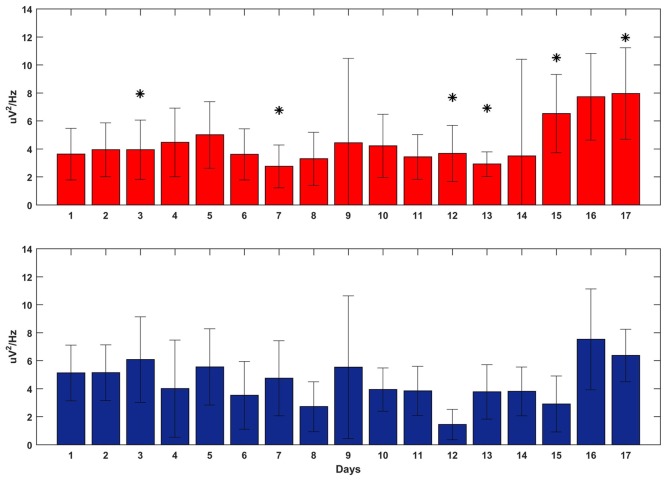
**Delta power at the reward onset during the execution of the BMI tasks overall 17 days of training (*x*-axis).** SWATS-area task (top panel, red), contra-area task (bottom panel, blue). Each bar represents the average delta power over all trials (mean ± STD). The * indicates the days when the difference in power between the two tasks was significant (*p*-value < 0.05, Bonferroni corrected).

Figure 7 shows the averaged delta power around the reward onset during feedback-on time for both tasks. The plot does not show any clear and significant trend that correlates with the BMI performance: the power of the SWATS-area task was significantly higher (*p* < 0.05, Bonferroni corrected) than the power of the contra-area task for days 12, 15 and 17, but at day 13 the order was inverted and days 14 and 16 did not show any significant difference. Although the power generally increased for both tasks throughout the experiment (the independent variable “day” predicted the power data through a linear model with a *p*-value = 1.61e-74), the same model returned a *p*-value of 0.013 when the independent variable “task” was used to predict the same data. This outcome suggests that the improvement in BMI performance for SWATS area-task was mostly associated with an increase in the number of successful targets achieved per minute; the monkey learned to achieve more targets by generating oscillations with increased frequency but not of increased amplitude (Figures 6, 7).

### Task-Related Neural Signal

**Figure 8:**
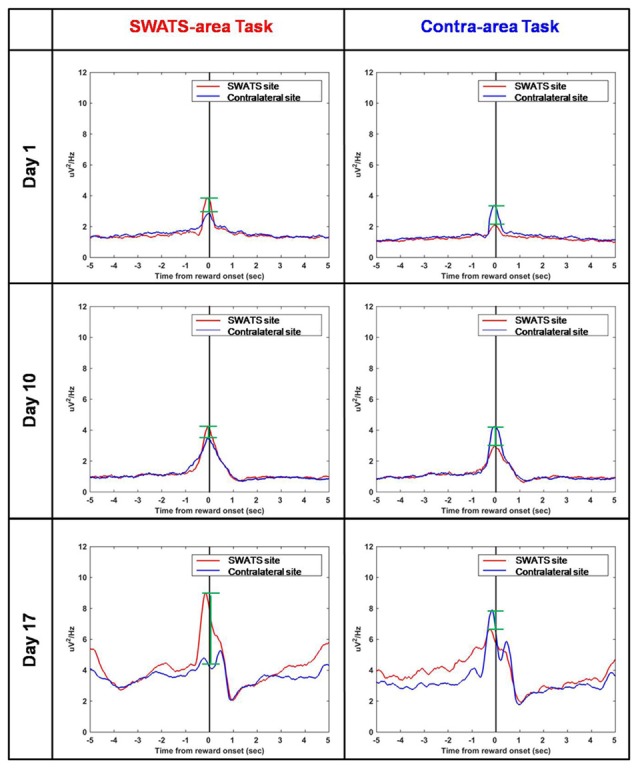
**Reward-triggered average of delta power for day 1 (top), day 10 (middle) and day 17 (bottom) during SWATS-area task (left) and contra-area task (right).** Red lines show the signal from SWATS electrode (conditioned hemisphere) and blue lines show the signal from contralateral electrode (non-conditioned hemisphere). X axis represents time from reward onset (black line). The green bars show the difference between the peaks of the two signals at the reward onset.

Figure 8 shows the reward-triggered average of delta power during the execution of both tasks for three representative days of training: day 1, day 10 and day 17. For each day, four conditions are shown: right site during the SWATS-area task, left site during contra-area task (in both cases, the signals were volitionally controlled), and right site during contra-area task, left site during SWATS-area task (non-volitionally controlled signals). There was no difference in the absolute value of delta power at the reward onset between SWATS-area task and contra-area task for the actively controlled electrode. Activation of the rewarded site was larger, even though left/right activations were not completely independent (i.e., during SWATS-area task delta power of contra-lateral electrode showed a peak at the reward onset and vice versa). This suggests that during training the monkey was volitionally controlling delta power preferentially on the electrode involved in the task.

## Discussion

Our findings indicate that delta-coupled stimulation during SWS may affect the learning rate of a motor cortex-dependent task. We used the Neurochip2 to deliver cortical stimuli precisely-timed to the depth-negative phases of SWS during nights following the training of a monkey in two BMI tasks. We found that the BMI task whose associated cortical site received stimulation during SWS (the SWATS site) was recalled easily and at a faster rate than the task in the hemisphere that received no stimulation.

### Slow-Wave Sleep and Off-Line BMI Learning

A night’s sleep is subdivided into REM and NREM periods based on specific neocortical rhythms, electrooculogram (EOG) and usually electromyogram (EMG; Kemp, 2010). REM exhibits mostly desynchronized neural activity, very similar to that observed during the waking state, while NREM sleep exhibits slow and widespread oscillations (Kemp, 2010). Using the head-fixed Neurochip2, we discriminated between sleep stages in a non-restrained macaque monkey by using three signals: head/whole body acceleration, ECoG and EOG (Figure 3). The NREM/ REM sleep cycle has a period of 60–90 min (Daley et al., 2006; Hsieh et al., 2008) and our overnight recordings showed this characteristic sleep architecture (Figure 3).

In our recordings, delta oscillations (1–4 Hz) of NREM SWS showed a significantly lower rate during wakefulness (Table 1) and tended to occur at discrete times throughout the night (Figures 3A, 4A). Importantly, this sleep structure was maintained when cycle-triggered stimulation was delivered, meaning that the stimulation did not alter the sleep stage cycles (Figure 4A). On nights when stimulation was delivered the rate of detected oscillations was, on average, lower than during nights without stimulation-possibly because the stimulus artifact which saturated the signal for around 300 ms, precluded detection of waves that occurred during that time (Figures 4B,C). The fact that slow wave-triggered stimulation did not alter the cyclic structure of sleep is an important point since REM and NREM stages have complementary roles in off-line learning (Frankland and Bontempi, 2005) and SWS is considered a key stage for memory consolidation (Peigneux et al., 2004; Ribeiro et al., 2004; Genzel et al., 2014).

Slow wave oscillatory cycles consist of depolarizing and hyperpolarizing states, which respectively represent global neuronal activation and global neuronal inactivation (Steriade et al., 1993a,b; Steriade, 2003; Steriade and Timofeev, 2003; Crochet et al., 2006). This neocortical rhythm is thought to coordinate interactions between the neocortex and subcortical structures necessary to integrate newly encoded memories with pre-existing long-term memories (Sirota et al., 2003; Sirota and Buzsáki, 2005; Genzel et al., 2014). The amount of SWS correlates with improvements in task performance (Huber et al., 2004; Pugin et al., 2015), suggesting that SWS may support acquisition of new skills. Gulati et al. (2014) showed that successful learning of a BMI task in rats correlates with an increased entrainment of the activity of task-related neural ensembles to the negative phase of SWS. Conversely, poor learning sessions were associated with no increase in SWS phase-locking, suggesting that new learning and skill acquisition are linked to a coherent re-activation of emergent task-related activity during post-learning SWS. Their finding showed that neuroprosthetic learning, like other procedural learning, goes through the same off-line processing to stabilize and consolidate the “prosthetic memory”. Therefore, to achieve good BMI control, mechanisms of long-term cortical plasticity have to take place.

Our study aimed to further investigate this hypothesis, by using closed-loop phase-dependent stimulation during sleep and by observing its behavioral consequences through performance of two BMI tasks in which the subject was being trained in parallel. Compared to a standard motor task, a BMI task is better controlled by the investigator, such that the task’s complexity can be easily adjusted to fit the experimental needs. Moreover, neural activity involved in the task is spatially localized, so cortical modifications related to the acquisition of new skills can be monitored. In our study, task performance was assessed by comparing the rate of success achieved during volitional control (feedback-on) to the spontaneous target acquisition rate (feedback-off). Besides day-to-day variability, both tasks showed a similar rate of “success” during feedback-off throughout the experiment, indicating that the two hemispheres were spontaneously generating the same amount of oscillatory activity (Figures 5A, 6). The amplitude of oscillations associated with volitional cursor control did not show the same increase as the BMI performance itself (Figure 7); this suggests that the monkey learned to increase not the amplitude of oscillations but rather the frequency of their occurrence, in order to obtain more rewards per minute. As shown in Figure 8, the monkey also learned to dissociate the two tasks by gaining active control of the neural signal between the two cortical sites, indicating that the monkey learned the two tasks in parallel. At the beginning of training, performance of both tasks was under chance level (i.e., feedback-off > feedback-on), but performance of the SWATS-area task started to significantly improve at day 12 of training and success rates were maintained above chance level until the end of the experiment. In contrast, performance of the contra-area task did not show a significant improvement until day 15 (Figure 6). At the end of training the performance of the SWATS-area task during feedback-on were significantly larger than those of contra-area task (Figures 5, 6). Thus, the monkey started to learn the contra-area task few days later than the SWATS task and even then its performance was not as good.

A possible interpretation of this finding is that the task controlled by the cortical site that received cycle-triggered stimulation during SWS was learned faster than the task controlled by the site with no conditioning stimulation. In this perspective, the stimulation is a direct cause of the sped up learning rate. However, the effect could simply be due to a difference between the difficulties of the two different tasks. In the same way that the brain has a finer control of one hand than the other (handedness), it could be possible that it was easier for the monkey to volitionally modulate the SWATS area compared to the contra-area. To our knowledge, a brain laterality effect in BMI control has not been documented in the literature. As we showed through the day-by-day feedback-off performance, the two tasks showed a similar baseline activity, which did not indicate a spontaneously preference for the SWATS task. Although this observation is relevant, it cannot be considered a conclusive measurement of task difficulty. To address this issue, a possible control experiment would be to show that the monkey can achieve the same BMI performance for both hemispheres without any stimulation.

### SWS-Triggered Stimulation and Synaptic Plasticity

There are two main hypotheses regarding the mechanisms underlying memory consolidation during sleep. The synaptic* homeostasis model* (Tononi and Cirelli, 2003, 2006) proposes that sleep promotes consolidation by global synaptic downscaling (depression). The active system *consolidation model* (McClelland et al., 1995; Marshall and Born, 2007) proposes that memory consolidation is the result of re-activation of memory traces during sleep. However these two hypotheses are not mutually exclusive and our study together with the observations of Gulati et al. (2014) might suggest that the two processes act in concert.

The homeostatic hypothesis of sleep is supported by both electrophysiological and molecular evidence showing that wakefulness is associated with net synaptic potentiation whereas sleep preserves the overall balance of synaptic strength through global synaptic depression (Vyazovskiy et al., 2008a,b). According to the homeostatic model the amount of local SWS is tied to the amount of synaptic potentiation that has occurred in a specific brain area during previous wakefulness, associated with training in the new task (Ghilardi et al., 2000; Huber et al., 2004). A possible mechanism that links local synaptic potentiation during wakefulness with the amount of SWS may be a strong activation of sodium-dependent potassium currents during SWS, due to the potentiation of cortico-cortical synapses during wakefulness. SWS comprises a depolarized up-state, during which single neurons fire at relatively high rates, followed by the hyperpolarizing down-state generated by an activation of sodium-dependent potassium current. Thus, potentiation of cortico-cortical connections would in turn lead to a longer and more hyperpolarized down state, therefore increased amplitude of slow oscillations (Amzica and Steriade, 1995; Steriade, 2003; Hill and Tononi, 2005). Slow waves of larger amplitude in the electroencephalogram are caused by an increase of slow oscillations at the single cell level together with an increase of synchronization of oscillations among neuronal populations.

Whichever the specific mechanism, the suggested role of sleep is to scale down synaptic strength in order to restore the neural circuits to a level energetically suitable by down-selecting or pruning some synapses (Tononi and Cirelli, 2003, 2006). As illustrated in recent studies, one way to do so is for neurons to reduce synaptic strength in an activity-dependent manner, which protects against depression the synapses that are strongly activated during previous wakefulness and down-selects the synapses that are weakly activated (Hashmi et al., 2013; Nere et al., 2013). Thus, during sleep, stronger synapses, which presumably capture aspects of sensory experience that agree with previously acquired knowledge, survive and are consolidated, whereas weak synapses, which are more likely to reflect background noise, are depressed.

Overall, learning ability is de-saturated by maintaining a balance in the synaptic inputs of cortical neurons. In line with this model, Gulati et al. (2014) found that a “replay” of task-related activity is in phase with up-states of SWS. Our results show that delta-coupled stimulation in phase with SWS up-states facilitated learning of a BMI task over a second competitive one. Besides being a local phenomenon (Ghilardi et al., 2000; Huber et al., 2004, 2006), SWS has been described as a global phenomenon (i.e., occurring in phase across most brain areas), which allows concurrent reactivation of the newly encoded traces in different structures, including the hippocampus and neocortex, thereby serving to potentiate the cortico-cortical connections underlying stored representations (Logothetis et al., 2012; Miyamoto et al., 2016). Slow oscillations generate an important functional reorganization of cortical network that supports the consolidation of memories (Sirota et al., 2003; Sirota and Buzsáki, 2005; Genzel et al., 2014). In line also with the competitive down-selection principle (Hashmi et al., 2013; Nere et al., 2013), one interpretation of our result is that an enhancement of activity during the depolarizing up-states of SWS resulted in a potentiation of some synapses over the others. As a consequence, off-line processes underlying consolidation of the BMI task whose cortical site received SWS-dependent stimulation could have been facilitated and that task was more easily recalled during the daily training.

The evidence from this case study remains preliminary, so we cannot make a definitive conclusion about the mechanisms underlying memory consolidation nor claim that we enhanced learning. Several control experiments are needed to address open questions. For example, what is the role of up and down states of slow oscillations in off-line learning? Would stimulation triggered by the SWS hyperpolarizing down-states slow down the learning rate of the task? How does the stimulation frequency affect the learning process? Would randomized stimulation, i.e., not in-phase with any oscillation, affect the off-line learning process? Can these effects be generalized to other BMI tasks which do not explicitly involve SWS?

While not providing a conclusive answer to these issues, this case study illustrates a novel and effective methodology to further investigate off-line learning during sleep; these techniques could be adopted to further elucidate mechanisms of cortical plasticity associated with brain oscillations.

## Author Contributions

IR, SZ designed the research and prepared figures; IR performed experiments and analyzed data; drafted manuscript; IR, SZ and EEF interpreted results of experiments and approved final version of manuscript. SZ and EEF edited and revised manuscript.

## Conflict of Interest Statement

The authors declare that the research was conducted in the absence of any commercial or financial relationships that could be construed as a potential conflict of interest.
